# Ultrasound vector flow imaging during veno-arterial extracorporeal membrane oxygenation in a thoracic aorta model

**DOI:** 10.1007/s10047-023-01413-z

**Published:** 2023-07-20

**Authors:** Kenichiro Yambe, Takuro Ishii, Billy Y. S. Yiu, Alfred C. H. Yu, Tomoyuki Endo, Yoshifumi Saijo

**Affiliations:** 1 Graduate School of Medicine, Tohoku University, 2-1 Seiryo-Machi, Aoba-Ku, Sendai, Miyagi 980-8575 Japan; 2 Department of Hepatobiliary and Pancreatic, Tohoku Medical and Pharmaceutical University, 1-15-1 Fukumuro, Miyagino-Ku, Sendai, Miyagi 983-8536 Japan; 3 Frontier Research Institute for Interdisciplinary Sciences, Tohoku University, 6-3 Aramaki Aza Aoba, Aoba-Ku, Sendai, Miyagi 980-8578 Japan; 4 Graduate School of Biomedical Engineering, Tohoku University, 6-6-05 Aramaki Aza Aoba, Aoba-Ku, Sendai, Miyagi 980-8579 Japan; 5 Research Institute for Aging, University of Waterloo, 250 Laurelwood Drive, Waterloo, ON N2J 0E2 Canada; 6 Division of Emergency and Disaster Medicine, Tohoku Medical and Pharmaceutical University, 1-15-1 Fukumuro, Miyagino-Ku, Sendai, Miyagi 983-8536 Japan

**Keywords:** VA-ECMO, Hemodynamics, Ultrasound flow imaging, Vector flow imaging, Thoracic aorta, Mixing zone

## Abstract

In veno-arterial extracorporeal membrane oxygenation (VA-ECMO) treatment, the mixing zone is a key hemodynamic factor that determines the efficacy of the treatment. This study aimed to evaluate the applicability of a novel ultrasound technique called vector flow imaging (VFI) for visualizing complex flow patterns in an aorta phantom under VA-ECMO settings. VFI experiments were performed to image aortic hemodynamics under VA-ECMO treatment simulated in an anthropomorphic thoracic aorta phantom using a pulsatile pump (cardiac output: 2.7 L/min) and an ECMO pump with two different flow rates, 0.35 L/min and 1.0 L/min. The cardiac cycle of hemodynamics in the ascending aorta, aortic arch, and descending aorta was visualized, and the spatio-temporal dynamics of flow vectors were analyzed. VFI successfully visualized dynamic flow patterns in the aorta phantom. When the flow rate of the ECMO pump increased, ECMO flow was more dominant than cardiac output in the diastole phase, and the speed of cardiac output was suppressed in the systole phase. Vortex flow patterns were also detected in the ascending aorta and the arch under both ECMO flow rate conditions. The VFI technique may provide new insights into aortic hemodynamics and facilitates effective and safe VA-ECMO treatment.

**Supplementary Information:**

The online version contains supplementary material available at 10.1007/s10047-023-01413-z.

## Introduction

Veno-arterial extracorporeal membrane oxygenation (VA-ECMO) treatment is performed for patients with severe cardiopulmonary failure in order to maintain blood flow to the patient’s organs. Various factors, such as cardiac output, vascular resistance, flow rate of the ECMO pump, and cannula diameter, interactively change the hemodynamics in the aorta, and even slight changes in parameters may cause a rapid deterioration of the patients’ vital signs [1]. Therefore, the procedure requires intensive and precise control of these parameters.

One of the key hemodynamic factors that indicate the efficacy of VA-ECMO treatment is known as the location of the “mixing zone,” where cardiac output flow meets the retrograde ECMO flow supplied through a cannula in the femoral artery [1, 2]. When VA-ECMO is introduced to a patient and their native cardiac output is severely low, the mixing zone must be controlled to be around the aortic arch (hereafter referred to as the arch) in order to maintain sufficient oxygen supply to the patient’s brain. In contrast, when native cardiac output is recovered, the mixing zone must be in the descending aorta by reducing the ECMO flow rate to avoid an increase in left ventricular afterload [3]. As such, the location of the mixing zone has been important for optimizing ECMO flow setting and reducing the risk of complications, such as hypoxic encephalopathy and heart failure [2]. In addition, VA-ECMO treatment often causes severe complications such as thromboembolism and bleeding caused by coagulopathy. Reports have shown that 5.9% and 40.8% of patients who underwent ECMO treatment had cerebral infarction and bleeding complications, respectively [4], which may be related to cerebrovascular accidents and postoperative cognitive dysfunction [5]. Although multiple factors are involved in these complications, it is evident that nonlaminar blood flow dynamics in the thoracic aorta is one of the important risk factors that affect coagulability of the blood [6]. Despite the importance of assessing hemodynamics in the aorta, in the present clinical practice, there is no monitoring method established for directly observing and evaluating the mixing zone and associated flow dynamics in the thoracic aorta, which have been roughly estimated by measuring oxygen partial pressure (PO_2_) at the right radial artery and cerebral regional saturation of oxygen (rSO_2_) [7, 8].

To investigate the transition of the mixing zone in the aorta, computational flow dynamics simulation is not desirable as it lacks the point-of-care diagnosis capability that is necessary for adjusting VA-ECMO treatment parameters. In addition, cine magnetic resonance imaging (MRI) is not applicable for patients undergoing VA-ECMO treatment [9]. Alternatively, color Doppler imaging using a transesophageal echocardiography (TEE) may be a potential solution because it has real-time imaging capability and can be used under VA-ECMO treatment [10, 11]. Nevertheless, while color Doppler imaging is valid for assessing the mixing zone in the abdominal aorta [12], to the best of our knowledge, color Doppler imaging has not been used to identify the mixing zone in the thoracic aorta due to the limited spatial and temporal resolvability of present color Doppler imaging, which is not sufficient for imaging the mixing zone in the curved thoracic aorta. Furthermore, although there is an ultrasound quantitative flow imaging technique known as vector flow mapping (VFM) [13], the application of this modality has been practically limited to visualizing flow dynamics in the left ventricle due to its geometrical assumption in the VFM algorithm.

To overcome the limitations of the present modalities, a novel ultrasound flow imaging technique, called vector flow imaging (VFI), may be useful [14–19]. VFI is based on high-frame rate ultrasound imaging techniques and can visualize complex flow dynamics in the body by displaying the spatio-temporal distribution of both flow direction and speed beyond > 1,000 frames per second (fps). To date, VFI techniques have been tested in complex flow scenarios such as those in the carotid bifurcation [18–20] and the prostatic urethra [21, 22] and have shown strong potential to provide new insights into their pathophysiological understanding; however, they have not yet been assessed in the visualization of the flow in the thoracic aorta, especially under VA-ECMO treatment conditions. Therefore, this study hypothesized that VFI can be used to visualize the changes in spatio-temporal hemodynamics around the mixing zone in the thoracic aorta at different ECMO flow conditions.

To spearhead this research direction, this study aimed to test the hypothesis on a bench-top condition by visualizing flow dynamics under VA-ECMO treatment simulated in an anthropomorphic thoracic aorta phantom using a research-purpose ultrasound VFI framework. A thoracic aorta phantom system was devised to simulate the blood flow in the aorta under ECMO treatment, and the dynamic aorta flow in two different ECMO flow settings was imaged and visualized with a VFI system. Finally, the feasibility of VFI in observing the mixing zone was discussed.

## Materials and methods

### The thoracic aorta phantom and aortic flow system

**Fig. 1:**
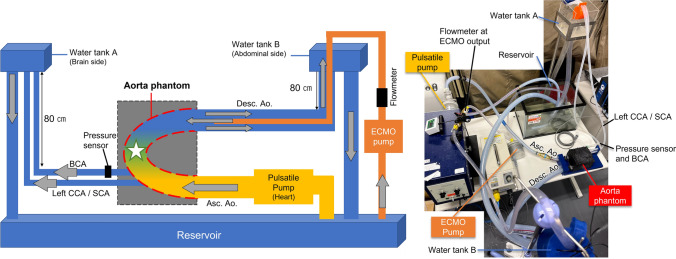
Schematic diagram (left) and photograph (right) of the thoracic aorta phantom and flow system

**Fig. 2:**
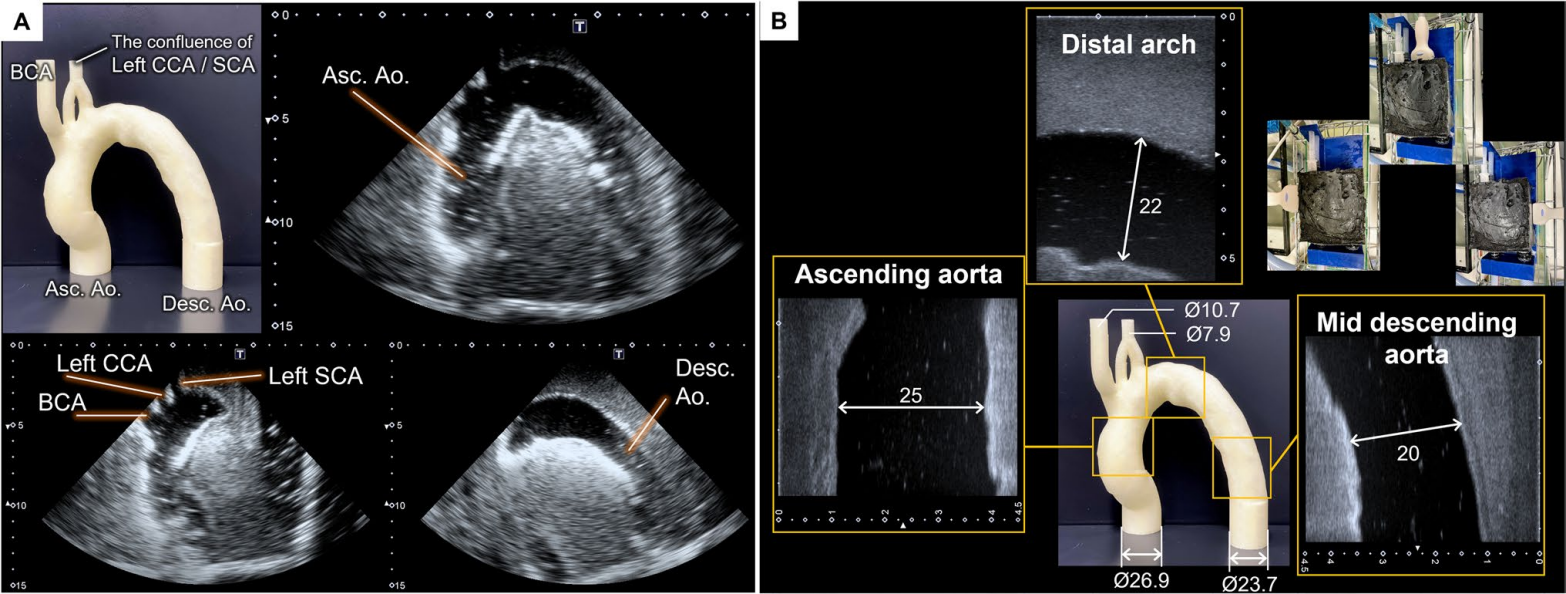
**A** The thoracic aorta model printed with a 3D printer and ultrasound images of the aorta phantom acquired with a sector probe. **B** B-mode images of the aorta phantom taken with a linear array probe. On the upper-right side, the location of the probe for each measurement is shown. The diameters are shown in mm

To conduct ultrasound VFI experiments, an anthropomorphic phantom of the human thoracic aorta and a flow system to simulate hemodynamics in the aorta under VA-ECMO treatment was developed (Fig. 1). The thoracic aorta phantom illustrated in Fig. 1 (hereafter referred to as the aorta phantom) was designed and fabricated based on a protocol of polyvinyl alcohol (PVA) wall-less phantoms [23] so that it resembles the internal geometry of the human thoracic aorta that was extracted from anonymized contrast-enhanced computed tomography images (Fig. 2 A). The use of human-origin data was approved by the local ethics committee at graduate school of engineering, Tohoku University. The fabricated phantom had four outlets: the ascending aorta (Ø26.9 mm), the brachiocephalic artery (BCA, Ø10.7 mm), the confluence of the left common carotid and left subclavian arteries (Ø7.9 mm), and the mid descending aorta (Ø23.7 mm). The fabrication process of the aorta phantom is described in Supplementary Content 1. In addition, as shown in Fig. 1, a flow system was built to generate complex flow dynamics, such as the mixing zone, in the aorta phantom. The ascending aorta inlet was connected to a cardiac pulsatile pump (A mechanical piston pump with two ball valves; valve and outlet diameter: 15.8 mm; 55-3305, Harvard Apparatus, MA, USA) via a 90 cm tube (inner diameter: 15.8 mm), while the descending aorta outlet and the aortic trifurcation outlets were connected to water tanks, which were placed at a height (80 cm) relative to the aorta phantom to mimic diastolic blood pressure. Blood pressure in the phantom was measured at the BCA outlet using a pressure monitoring system (TSD104 and MP160 system, BIOPAC Systems Inc., CA, USA). Furthermore, in order to simulate retrograde ECMO flow in the phantom, a centrifugal pump for ECMO treatment (hereafter referred to as the ECMO pump; CAPIOX SP-101, Termo Corp., Tokyo, Japan) with an outlet flow meter (DigiFlow 6710 M, KRONE Corp., Tokyo, Japan) was used. Note that no artificial lung module was connected to the system. A tube (inner diameter: 12 mm) from the ECMO pump outlet was inserted into the flow circuit without cannulas as shown in Fig. 1, so that the tip of the tube was located at the outlet of the descending aorta of the phantom. Finally, a blood-mimicking fluid consisting of 10% aqueous solution of glycerin (072-00621, FUJIFILM Wako Pure Chemical Corp., Osaka, Japan) (density: 1.02 kg/m^3^) and 0.5% of 8.0–13.0 μm silica particles (density: 2.1 kg/m^3^; Godd ball B-25C, Suzukiyushi Industrial Corp., Osaka, Japan) was used as the fluid to circulate in the aorta phantom to mimic the density and acoustic properties of human blood [24, 25]. Although the density of silica particles was higher than that of fluid, the negligible sedimentation velocity of the particles due to their small diameter ensures that this property does not affect the flow investigations conducted in this study.

To simulate the hemodynamics in the aorta phantom with a mixing zone at different locations, two ECMO flow rate settings were used in this study: low (0.35 L/min), and high (1.0 L/min). For both ECMO conditions, the same cardiac output (stroke volume: 30 mL; heart rate: 90 bpm; cardiac output: 2.7 L/min, systole/diastole ratio: 40/60%) was used for the pulsatile pump. In this pulsatile pump setting, the blood pressure at the BCA without ECMO flow was measured to be 81/57 mmHg, which was similar to low output syndrome (stroke volume < 40 mL) [26]. The flow rate at the outlet of the trifurcation aorta was measured to be 1.3 L/min in low ECMO flow and 1.7 L/min in high ECMO flow, respectively. Prior to VFI experiments, conventional pulse Doppler imaging in the aorta phantom was performed to show the basic flow properties in the phantom and the images are shown in Supplementary Content 2.

### Ultrasound vector flow imaging

Imaging of the flow dynamics in the aorta phantom under two VA-ECMO conditions was performed using a VFI framework, so-called vector projectile imaging (VPI) [16]. To perform VPI measurements in the experiment, a research-purpose ultrasound platform (Vantage 256, Verasonics Inc., WA, USA) equipped with a 5 MHz linear array transducer (L11-5v, Verasonics Inc.) was utilized. The linear array probe was placed at three positions corresponding to the ascending aorta, distal arch, and mid descending aorta in the phantom (Fig. 2B) to image the flow dynamics in the entire thoracic aorta. The path length from the root of ascending aorta to those three imaging positions were approximately 50, 110 and 180 mm, respectively.

**Fig. 3:**
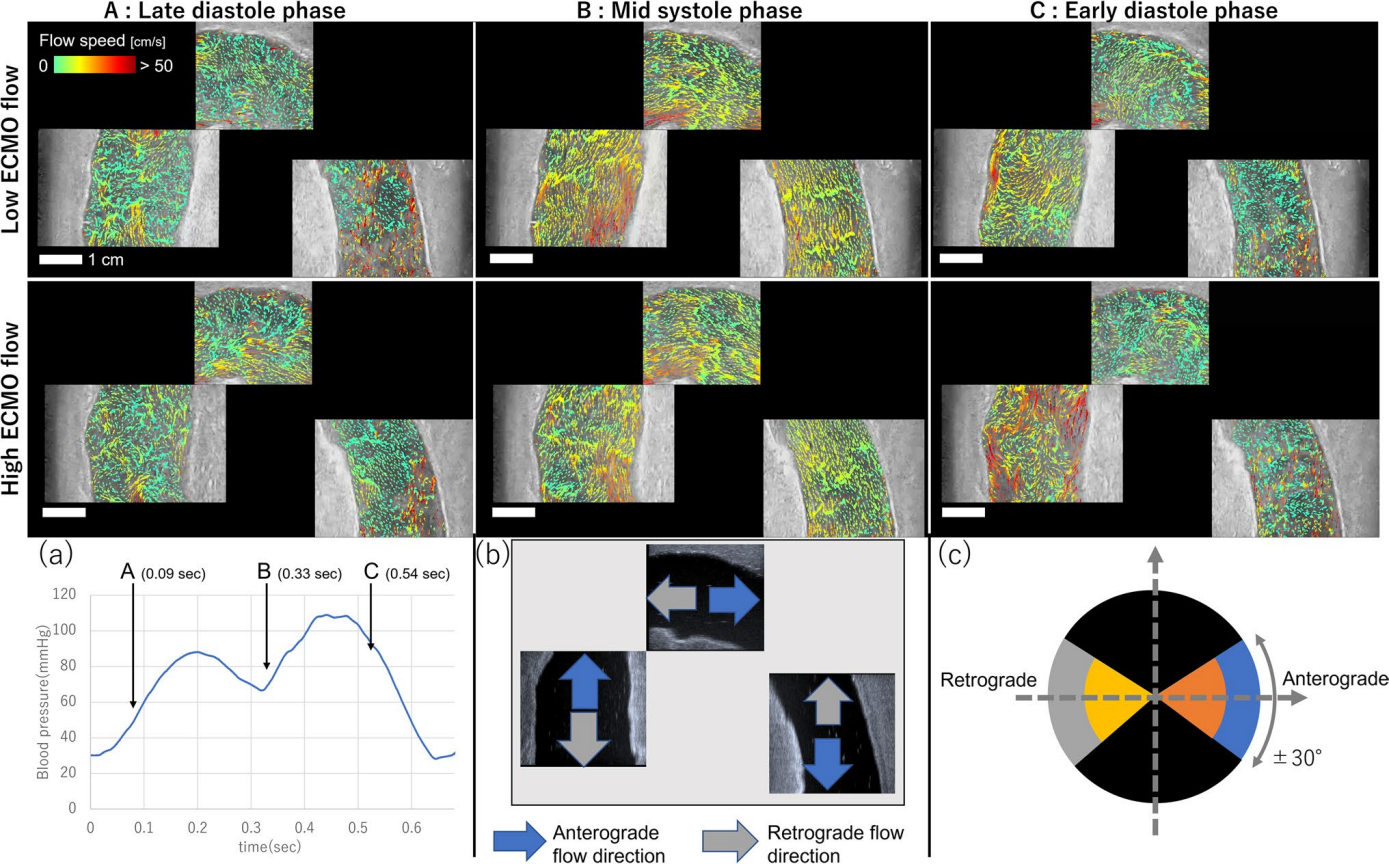
Screenshots of the VPI cineloops of low ECMO flow condition (upper row) and high ECMO flow condition (lower row). The timing of each image corresponds to the A, B, and C labels in **a** showing a blood pressure waveform. **b** Definition of anterograde and retrograde flow directions for each image. **c** Range of anterograde and retrograde flow vectors. Speed of blue and gray region is above 0.25 m/s

The detailed principle and algorithm of the flow vector estimation and VPI framework can be found in [15, 16]. In brief, the ultrasound system was configured to emit plane-wave ultrasound pulses from two different transmission angles (−10° and 10°) alternatively at a pulse repetition frequency of 10 kHz in order to simultaneously acquire two color-Doppler cineloops, which correspond to the Doppler shifts of each transmission angle. Those multiple color Doppler data was used to construct a linear equation based on Doppler equation to derive flow vectors (i.e., flow direction and speed at each pixel in each frame). Finally, the flow vectors were estimated by solving the linear equation with the least-squares method [15]. The final flow vector data was visualized by a dynamic vector rendering technique proposed in the VPI framework. In this study, for each ECMO flow condition, three VPI cineloops (respectively, acquired from three probe positions) were obtained. Each cineloop visualized a region of 38 mm (width) × 40 mm (depth) from the probe for a duration of 1.5 s that involved at least one cardiac cycle of flow data. These three cineloops were synchronized and synthesized into one video file (Fig. 3A-C) using the blood pressure waveforms (Fig. 3a) recorded at the BCA simultaneously in each VPI measurement.

Using the time in the blood pressure curve (Fig. 3a), a cardiac cycle (approx. 0.66 s) of VPI data was divided into three phases: late diastole (< 0.1 s), systole (0.1–0.4 s), and early diastole (> 0.4 s). In our experiment setting, it was not possible to determine the start time of the systolic action of the cardiac pump. In addition, the pressure curve showed a bi-modal shape due to incomplete (e.g., bouncing) ball valve actions in the cardiac pump that made identifying the systole phase difficult. Therefore, in this study, we defined the start time of the systole as that of the first observation of the anterograde flow vectors in the proximal end of the ascending aorta images (at 0.1 s) and the systole duration as 0.3 s from the pressure curve observation.

### Analyses of the visualized flow vectors

The visualized flow vectors in the thoracic aorta were further analyzed to assess temporal changes in the flow vector distribution. For this purpose, the flow vectors were categorized into five groups: fast anterograde, slow anterograde, fast retrograde, slow retrograde, and other flow vectors. For each imaging position, the anterograde and retrograde flow directions were defined as the flow direction from the pulsatile pump and the ECMO pump, respectively (Fig. 3b). For each VPI cineloop, flow vectors directed to ± 30° with respect to the anterograde or retrograde direction were categorized into the anterograde flow group or the retrograde flow group, and the other flow vectors were placed in the other flow vectors group. In addition, the flow vectors in the anterograde and retrograde flow groups were further divided into the fast or slow group using a threshold of 0.25 m/s (Fig. 3c). After categorization, the area of each flow group was counted for each frame, and the ratio of each flow group with respect to the flow area in the frame was calculated. To compare the ratio of respective flow groups between two ECMO flow conditions, mean and standard deviation of those ratio values in each cardiac phase (i.e., late diastole, systole, or early diastole) were derived for respective imaging positions and ECMO flow conditions. Welch’s t-test was used for the statistical analyses.

## Results

The VPI framework clearly visualized transient flow dynamics in the thoracic aorta in both ECMO pump conditions at 1250 fps (800 μs/frame). Figure 3A–C shows frames of the generated VPI cineloops in late diastole (0.09 s), systole (0.33 s), and early diastole (0.54 s), respectively.

In the VPI cineloops (Supplementary Content 3), various flow dynamic features associated with hydrodynamic interactions between cardiac output and ECMO flow were observed in different phases of the cardiac cycle. As designed in the experiment, we observed that the retrograde ECMO flow in the high ECMO flow condition and its mixing zone moved to the ascending aorta (Fig. 3A), while the native cardiac flow in the mid-systole phase was suppressed in the descending aorta compared to that in the low ECMO flow condition (Fig. 3B). In addition, the high-temporal resolvability of VPI effectively visualized the transition of ECMO flow from the descending aorta to the arch and ascending aorta in the diastole phase. In particular, in the transitional periods such as late and early diastole phases, the flow patterns of both conditions were more turbulent (Fig. 3C).

### Temporal changes in flow direction and speed at different ECMO conditions

**Fig. 4:**
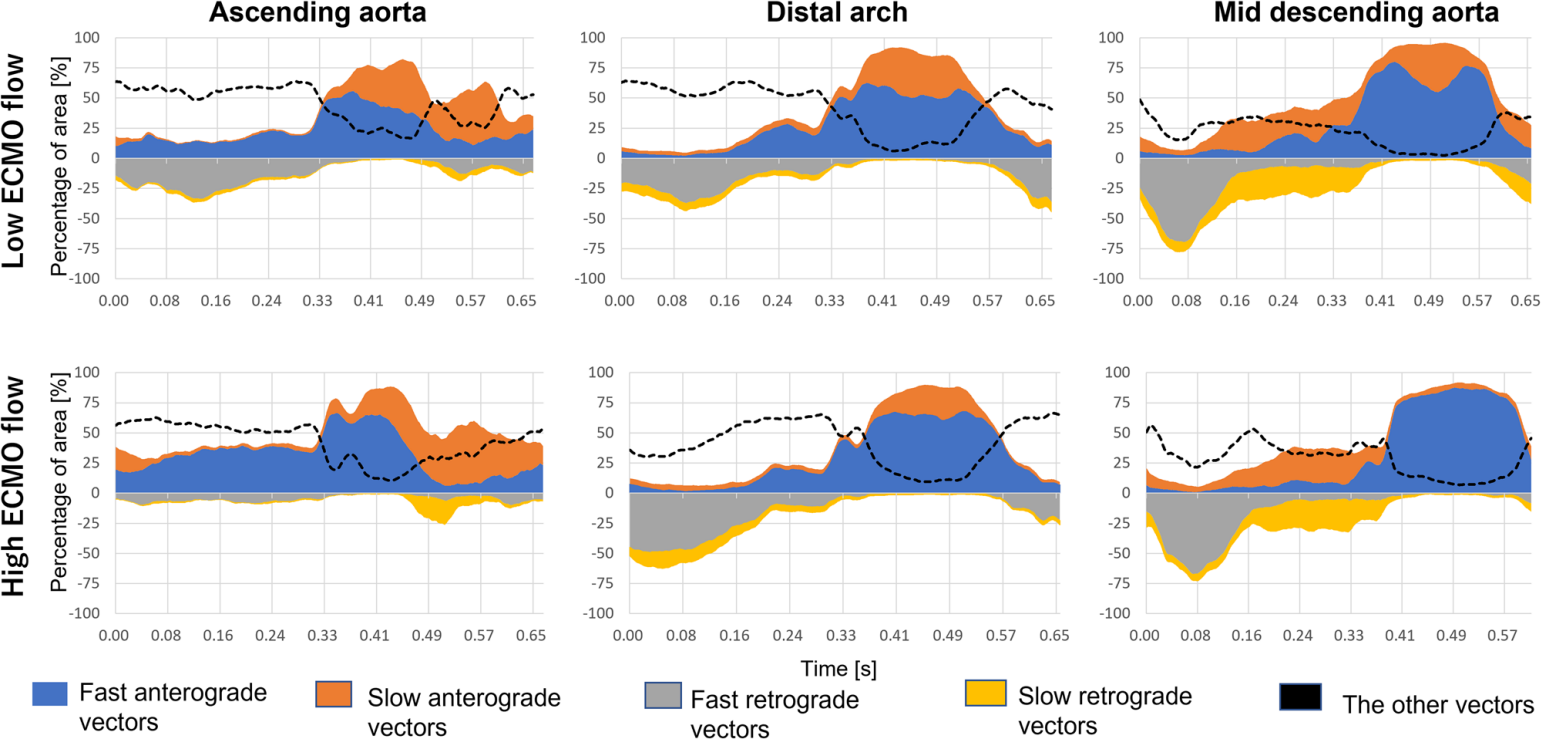
Time-resolved distribution of flow vector direction and speed

Observations in the previous section were quantified using the categorized flow vectors (Fig. 4). In late diastole, the ratio of retrograde flow vectors (i.e., ECMO flow) in the arch in the high ECMO flow was 59 ± 2.43% and higher than 33 ± 4.97% of the low ECMO flow (*p* < 0.01). Once the systole phase began, the anterograde native cardiac flow in the mid descending aorta spontaneously increased in the low ECMO flow. In the systole phase, the ratio of anterograde flow vectors of the low ECMO flow was 32 ± 23% (distal arch) and 40 ± 14% (mid descending aorta), which were significantly higher than 28 ± 21% and 32 ± 12% observed in the high ECMO flow, respectively (*p* < 0.01). In early diastole, anterograde flow gradually decreased and backed up with the retrograde direction flow from the ECMO pump in both conditions. Moreover, during the transition phase between systole and diastole (at around 0.33 and 0.57 s), the ratio of “the other vectors” group increased (especially in the arch), indicating an increase in turbulent flow components.

### Vortex flow features in the thoracic aorta

**Fig. 5:**
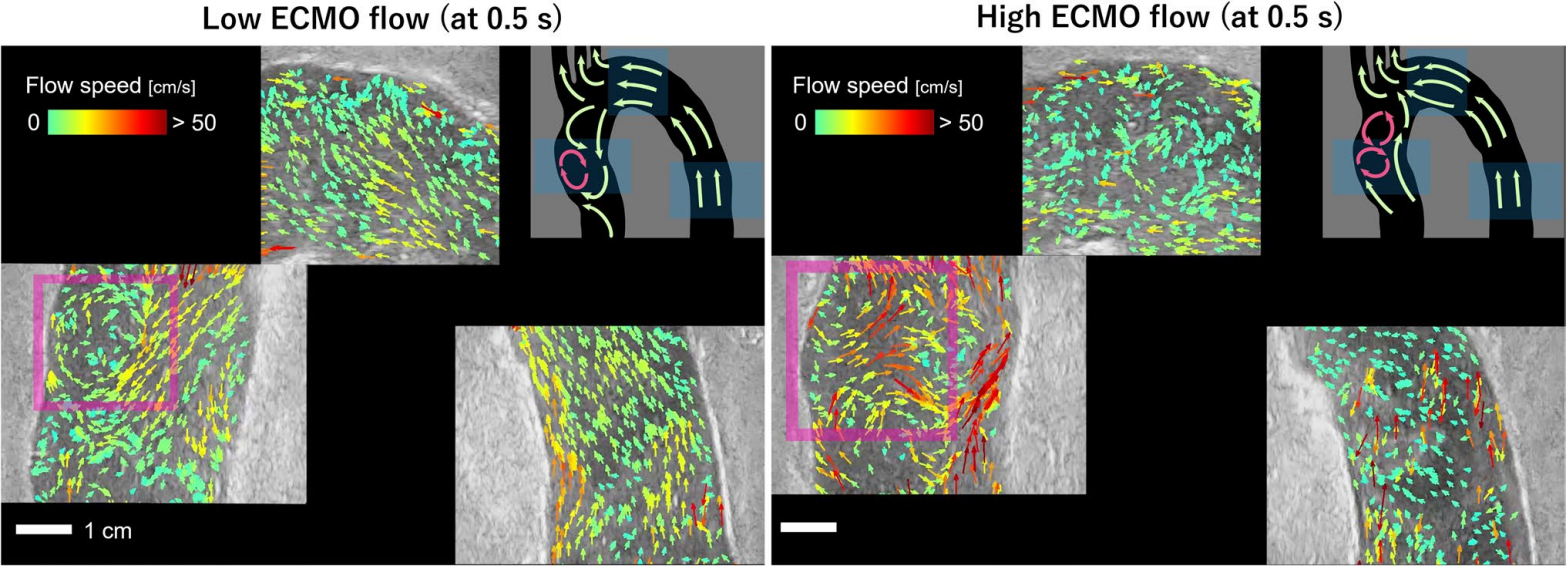
Vortex flow patterns observed in early diastole (at 0.5 s) in each flow condition. The VPI frames were generated with larger arrows at less concentration to enhance the visibility of the vortex features (in the pink rectangles). The sketch of the flow profile shows a possible flow pattern in the whole thoracic aorta. The location of the imaging position is labeled in blue and the observed vortex features are shown in pink color

The high spatio-temporal resolvability of the VPI technique could be used to observe the presence and transition of turbulent and vortex flow features in various time and position in the aorta phantom as shown in the VPI cineloops (Supplementary Content 3). For example, large vortex flow patterns were observed in the ascending aorta in the early diastole phase (at 0.5 s; Fig. 5). In the low ECMO flow condition, a vortex flow feature was observed to be caused as the ECMO flow collided with the wall of the ascending aorta and the output of the pulsatile pump. In addition, in the high ECMO flow condition, two vortex features were observed, suggesting an increase in the hydrodynamic interactions between the ECMO flow and the cardiac output.

## Discussion

The novel ultrasound VFI technique has been developed in order to unveil complex flow dynamics in the human body. Previous studies have shown the high accuracy of the flow vectors in both magnitude and angle estimated with VFI and its applicability to visualize multi-directional (bifurcation and vortex) flow patterns [15, 16]. Leveraging these foundations, this study showed that the VFI technique can also visualize the comprehensive hemodynamic events occurring in the thoracic aorta under an experimental setting of VA-ECMO treatment, effectively indicating when, where, and how the native cardiac and ECMO flow passed and mixed in the thoracic aorta. The visualized blood flow vectors showed differences in the location of the mixing zone between high and low ECMO flow rate visually (Fig. 3) and quantitatively (Fig. 4), as well as vortex flow patterns caused by interactions among the cardiac and ECMO streams and the vessel geometry (Fig. 5). Compared to conventional Doppler techniques that only measure one-directional flow speed along the ultrasound beam direction, VFI specifically enables the capture of multi-directional and high-transient flow patterns in the thoracic aorta and to quantify its spatio-temporal characteristics.

One of the potential clinical applications of this new ultrasound modality is to visualize the movement of the mixing zone that may aid in the safe guiding of the ECMO flow rate, especially while introducing and weaning ECMO treatment, detecting the risks of hypoxic encephalopathy and an excess increase in cardiac afterload [2]. Furthermore, the quantitative assessment of the vortex and nonlaminar flow feature may be applied to identify the location and the degree of the blood flow stasis that would increase risks of cerebral infarction and bleeding complications, which are known as major complications during ECMO treatment [4, 27].

To validate such diagnostic applicability, future studies must investigate VFI further in various aortic flow patterns, such as in different aorta sizes, mechanical properties, flow conditions, and imaging planes, because this study only measured flow in one aorta phantom at three imaging planes and with a limited combination of the native and ECMO flow rate. Also, the deformation of the aortic wall and the viscosity of the blood were not considered in the experiment. Those properties would affect pressure distribution and hydraulic interactions between native and ECMO flow in the aorta and might show different flow patterns in VFI. To take those properties into account, future studies should adjust the elasticity of the PVA phantoms [28, 29] and the viscosity of the blood-mimicking fluid [30]. In addition, the ECMO flow rates in this study (0.35 and 1.0 L/min) had to be lower than that of used in the actual VA-ECMO treatment (> 3 L/min) in order to observe the mixing zone at around the arch. This discrepancy may be attributed to the limitations in replicating the ECMO flow to the abdominal aorta due to (1) differences in the size and positioning of the ECMO flow outlet compared to the actual ECMO cannulas, and (2) non-physiological properties of the aorta phantom, such as the elasticity of the aorta wall and the constant peripheral pressure determined by the height of the reservoirs. Fine adjustment of those flow circuit components would simulate more realistic flow profile, especially in the descending aorta and the aorta trifurcation branches. Moreover, this study only used one cardiac flow condition as the ball valve installed in the cardiac pump did not precisely simulate the aortic valve motion and caused significant backward flow in the aorta when the flow rate of the pulsatile pump was increased. The cardiac pump system should be improved further to investigate aortic flow patterns with an increased cardiac output, for example during the weaning period of ECMO treatment.

Finally, to realize VFI measurements of the thoracic aorta in vivo, the development of a VFI system equipped with a TEE probe is necessary to increase the imaging depth and angle so that the imaging field can cover a wider area of the thoracic aorta, including the trifurcation branches, which could not be imaged with a linear array probe in this study. Since TEE probes have different acoustic transducer geometry from the linear array probes, a new imaging sequence for TEE-VFI system has to be designed using diverging wave imaging schemes [17, 19]. Also, a custom TEE probe that can be used with a research-purpose ultrasound platform is necessary to acquire raw echo signals to perform VFI processing. In clinical practice, TEE has already been utilized for monitoring patients undergoing ECMO treatment to adjust the position of the ECMO cannula, optimize the afterload of the left ventricle, and detect left ventricular thrombi [31]. Thus, when a TEE VFI system is developed, it will be readily combined with these present diagnostic techniques and will provide deep insights into the complex hemodynamics in the thoracic aorta under VA-ECMO treatment.

## Conclusions

This study aimed to perform the first attempt to test an ultrasound VFI technique on a bench-top condition to visualize the simulated hemodynamics in the thoracic aorta under VA-ECMO treatment. A bench-top thoracic aorta system was devised to simulate hemodynamics with two different ECMO settings, and the dynamic flow patterns were acquired using a VFI framework. The experiments successfully demonstrated that the VPI cineloop is capable of visualizing comprehensive flow dynamics caused by interactions between the ECMO flow and the cardiac output in various cardiac phases. The application of this technique to patients may provide new insights into aortic hydrodynamic conditions that aid effective and safe VA-ECMO treatments.

### Supplementary Information

Below is the link to the electronic supplementary material.

Supplementary file1 The fabrication protocol of the aorta phantom (PDF 724 KB)

Supplementary file2 Pulse Doppler imaging of flow in the aorta phantom (PDF 433 KB)

Supplementary file3 VPI cineloops of two cardiac cycles of aorta hemodynamics in each ECMO flow condition. The cineloops is first played in the actual time scale and then replayed at 1/10 slow-speed (MP4 20943 KB)

## Data Availability

The data that support the findings of this study are available from the corresponding author on reasonable request.
