# Supplementary material for: Ultrasound vector flow imaging during veno-arterial extracorporeal membrane oxygenation in a thoracic aorta model
Source: J Artif Organs. 2023 Jul 20;27(3):230–7. doi: 10.1007/s10047-023-01413-z (PMC11345325; doi:10.1007/s10047-023-01413-z)
Supplement: Supplementary file 1 — Supplementary file1 The fabrication protocol of the aorta phantom (PDF 724 KB) [file 10047_2023_1413_MOESM1_ESM.pdf]

Ultrasound Vector Flow Imaging During Veno-Arterial Extracoporeal Membrane Oxygenation in a Thoracic Aorta Model

Kenichiro Yambe, Takuro Ishii, Billy Y.S. Yiu, Alfred C.H. Yu, Tomoyuki Endo, Yoshifumi Saijo

Supplementary Content 1:
Fabrication Protocol of Thoracic Aorta PVA Phantoms

The aorta phantom was designed to have the internal geometry of the healthy human thoracic aorta, including the ascending and descending aorta and the aortic trifurcation. In this study, we assumed that the vessel wall is rigid and has acoustic properties of human tissue so that it can be imaged by ultrasound. To satisfy these requirements, we used a fabrication protocol for anthropomorphic wall-less phantoms reported previously[1,2].

First, three-dimensional (3-D) volume data of the thoracic aorta were constructed using ImageJ software[3] based on anonymized contrast-enhanced CT images (Fig. SC1-1A). The volume data were processed using Meshlab software (Fig. SC1-1B)[4] and Solidworks 2020 software (Fig. SC1-1C; Dassault Systems SolidWorks Corp., France) to extract the surface geometry of the thoracic aorta and to modify the shape of each outlet of the vessels to connect with the flow circuit. The diameters of the ascending and descending aorta outlets were respectively 26.9 and 23.7 mm, while those of brachiocephalic artery and the confluence of the left common carotid and subclavian arteries were 10.7 and 7.9 mm, respectively. As an example, cross-sectional dimensions measured at the ascending aorta, distal arch, and mid-descending aorta were 29 x 27 mm, 27 x 26 mm, and 24 x 22 mm, respectively. Those aorta sizes were considered to be the average size of that of Asians[5].

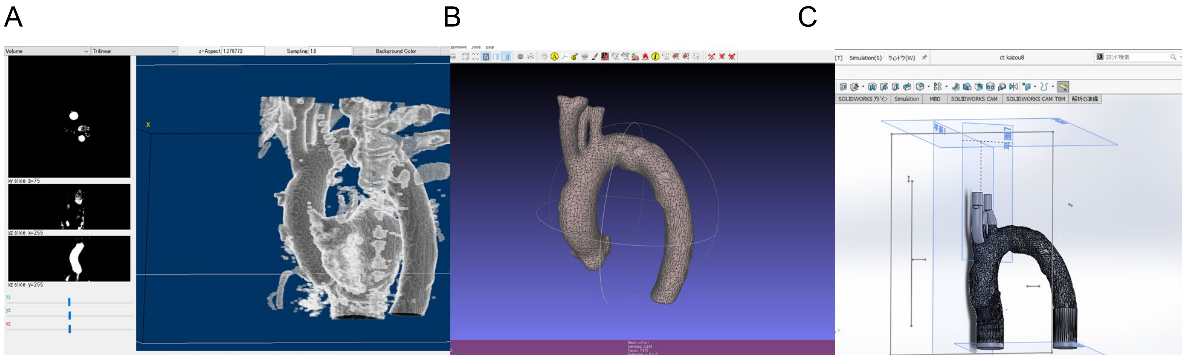

Figure SC1-1. The process of extracting aorta geometry from CT data

A: ImageJ software to reconstruct a 3D volume using vascular lumen CT values from contrast-enhanced CT images; B: Meshlab software to remove outlier components other than the arterial lumen; and C: Computer-aided design software (Solidworks2020) to modify the edges of the outlets so that they can be connected with the flow circuit.

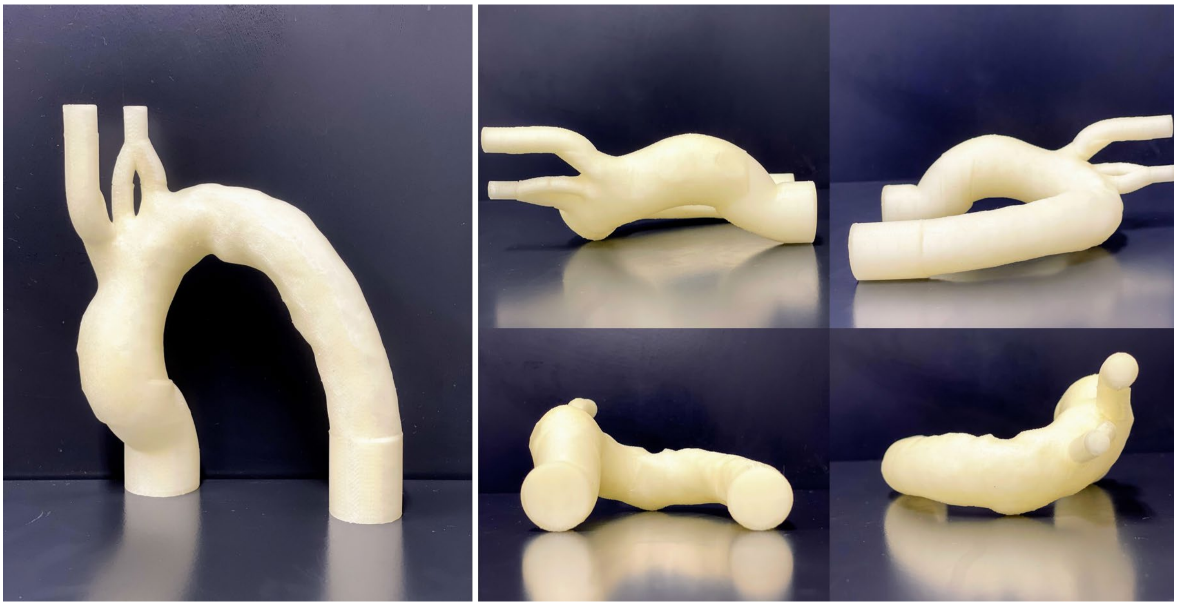

Figure SC1-2. The 3D-printed thoracic aorta model from multiple perspectives

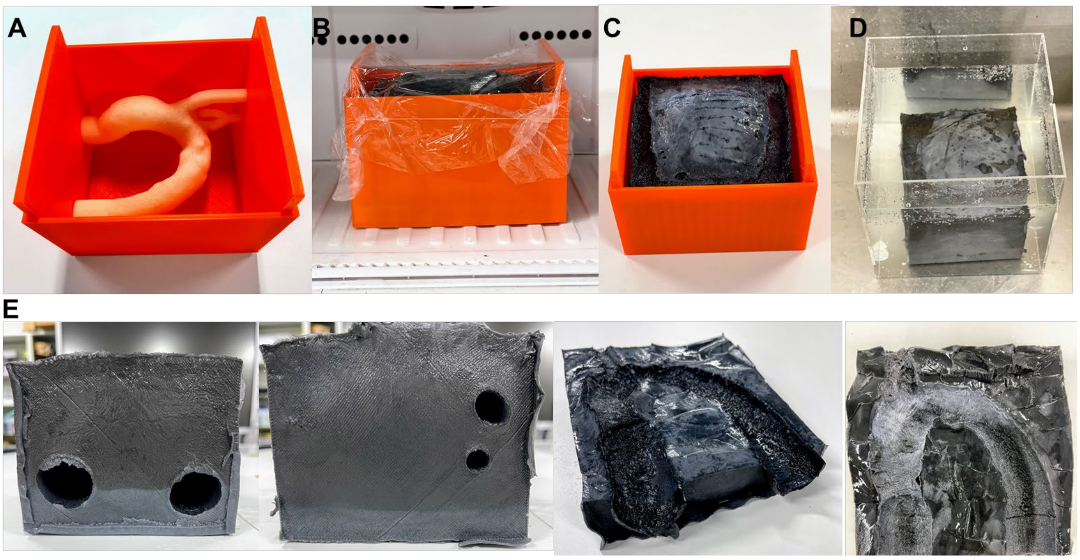

Figure SC1-3. Procedure for creating PVA phantom

A: The printed aorta model was placed in the mid-space of a casting box (inner dimension: 14 × 15 × 10 cm); BC: The PVA solution polymerized in four freeze–thaw cycles (frozen at - 20 °C for 24 h and thawed at 5 °C for 24 h); D: The entire box was submerged in a water bath for three hours to dissolve the aorta model. Since only the aorta model was water-soluble, the remaining PVA gel block contained a flow tract with the geometry of the thoracic aorta; and E: Photograph of the created PVA phantom and horizontal section.

Second, the thoracic aorta geometry was physically printed with a 3-D printer (UP300, Tiertime, Beijing, China) using a water-soluble polyvinyl alcohol (PVA) filament (MelFil, Nihon Gosei Kako Co., Ltd., Tokyo, Japan). The printed aorta model was placed in the mid-space of a casting box (inner dimension: 14 × 15 × 10 cm).

Third, the thoracic aorta model was assembled in the casting box and the gap between the model and the box was filled with an aqueous solution consisting of 15% PVA powder (341584, Sigma-Aldrich, MO, USA), 1.0% Graphite (282863, Sigma-Aldrich) as acoustic scatterer, and 0.3% potassium sorbate (85520, Sigma-Aldrich) as a preservative. After placing the casting box filled with the PVA solution in four freeze–thaw cycles (frozen at - 20 °C for 24 h and thawed at 5 °C for 24 h)[6], the entire box was submerged in a water bath for three hours to dissolve the aorta model. Since only the aorta model was water-soluble, the remaining PVA gel block contained a flow tract with the geometry of the thoracic aorta.

References

1. Ishii T, Yiu BYS, Yu ACH: Vector Flow Visualization of Urinary Flow Dynamics in a Bladder Outlet Obstruction Model. *Ultrasound Med Biol* 43: 2601-2610, 2017.
2. Ho CK, Chee AJY, Yiu BYS, Tsang ACO, Chow KW, Yu ACH: Wall-Less Flow Phantoms With Tortuous Vascular Geometries: Design Principles and a Patient-Specific Model Fabrication Example. *IEEE Trans Ultrason Ferroelectr Freq Control* 64: 25-38, 2017.
3. Schneider CA, Rasband WS, Eliceiri KW: NIH Image to ImageJ: 25 years of image analysis *Nat Methods* 9: 671-675, 2012.
4. Cignoni P, Callieri M, Corsini M, Dellepiane M, Ganovelli F, Ranzuglia G: MeshLab: an Open-Source Mesh Processing Tool, in: *Eurographics Italian Chapter Conference*. 2008, pp. 129–136.
5. Lee SH, Lee W, Choi HJ, Kim DJ, Park EA, Chung JW, Park JH: Measurement of the Aortic Diameter in the Asymptomatic Korean Population: Assessment with Multidetector CT, *J Korean Soc Radiol* 26: 105-112, 2013.
6. Xu H, Shi FK, Liu XY, Zhong M, Xie XM: How can multi-bond network hydrogels dissipate energy more effectively: an investigation on the relationship between network structure and properties. *Soft Matter* 16: 4407-4413, 2020.
