# Supplementary material for: Ultrasound vector flow imaging during veno-arterial extracorporeal membrane oxygenation in a thoracic aorta model
Source: J Artif Organs. 2023 Jul 20;27(3):230–7. doi: 10.1007/s10047-023-01413-z (PMC11345325; doi:10.1007/s10047-023-01413-z)
Supplement: Supplementary file 2 — Supplementary file2 Pulse Doppler imaging of flow in the aorta phantom (PDF 433 KB) [file 10047_2023_1413_MOESM2_ESM.pdf]

Ultrasound Vector Flow Imaging During Veno-Arterial Extracoporeal Membrane Oxygenation in a Thoracic Aorta Model

Kenichiro Yambe, Takuro Ishii, Billy Y.S. Yiu, Alfred C.H. Yu, Tomoyuki Endo, Yoshifumi Saijo

Supplementary Content 2:
Pulse Doppler Measurement of the Flow in the Aorta Phantom

To check the basic flow properties created in the aorta phantom, the flow speed in the aorta phantom was measured with the conventional pulse Doppler imaging method.

First, the aorta flow was created only using the cardiac pulsatile pump and no ECMO flow was delivered. The stroke volume and the heart rate of the cardiac pulsatile pump were 30 ml and 60 bpm, respectively. Fig. SC2-1 shows a pulse Doppler image of the ascending aorta. The maximum speed to the anterograde flow direction was measured to be 20.4 cm/s, which was in a range of blood flow speed at the left ventricle outlet (0 – 50 cm/s) in patients with lower output syndrome[1]. After the systole peak, the retrograde flow up to 11.3 cm/s was observed. The backward flow might be caused by incomplete ball valve actions (e.g., bouncing) in the cardiac pump.

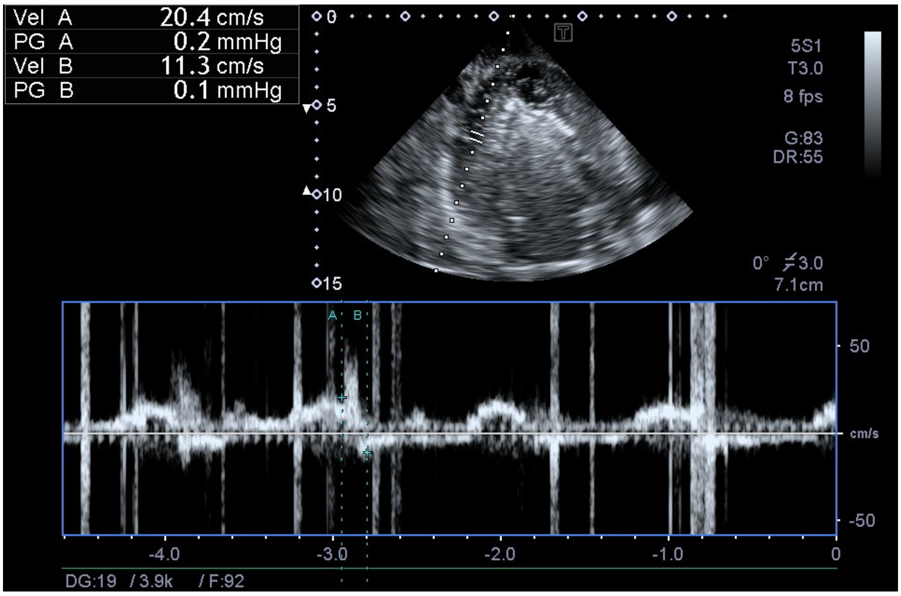

Figure SC2-1. Pulse Doppler imaging of the ascending aorta of the aorta phantom with only the cardiac pulsatile flow (Stroke volume: 30 ml).

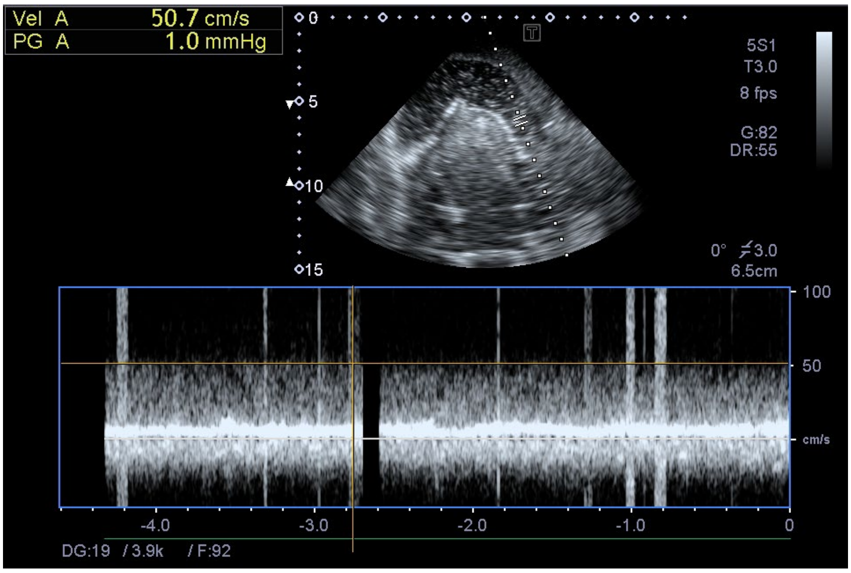

Figure SC2-2. Pulse Doppler imaging of the descending aorta of the aorta phantom with only ECMO flow (2.0 L/min).

Second, the cardiac pump was stopped and only ECMO flow (2.0 L/min) was created in the aorta phantom. Fig. SC2-2 shows a pulse Doppler image of the descending aorta in this condition. Since the ECMO pump creates a constant flow, the pulse Doppler image shows a constant flow feature, and the retrograde flow speed was measured to be 50.7 cm/s.

There was no leakage found in the thoracic aorta or the flow circuit. The flow rate at outlets of the trifurcation branch and the abdominal aorta (water tank A and B in Fig.1) in different ECMO pump settings were measured using a weight scale (Table SC2-1).

Table SC2-1. Flow rate at outlets in different ECMO flow settings

**Cardiac pump**	**ECMO pump**	**Flow rate at the trifurcation branch (Water tank A)**	**Flow rate at the abdominal aorta outlet (Water tank B)**
2.7 L/min (SV: 30 ml, 90 bpm)	0 L/min	0.5 L/min	2.2 L/min
	0.35 L/min	1.3 L/min	1.75 L/min
	1 L/min	1.7 L/min	2 L/min

References

1. Sawada K, Kawakami S, Murata S, et. al.: Predicting Parameters for Successful Weaning from Veno-Arterial Extracorporeal Membrane Oxygenation in Cardiogenic Shock, *ESC Heart Fail*, 8: 471-80, 2021.
